# Peripheral Vascular Resistance Impairment during Isometric Physical Exercise in Normotensive Offspring of Hypertensive Parents

**DOI:** 10.5935/abc.20170096

**Published:** 2017-08

**Authors:** Natália Portela, Josária Ferraz Amaral, Pedro Augusto de Carvalho Mira, Livia Victorino de Souza, Daniel Godoy Martinez, Mateus Camaroti Laterza

**Affiliations:** 1 Unidade de Investigação Cardiovascular e Fisiologia do Exercício - Faculdade de Educação Física e Desportos - Universidade Federal de Juiz de Fora, Juiz de Fora, MG - Brazil; 2 Disciplina de Nefrologia - Faculdade de Medicina - Universidade Federal de São Paulo, São Paulo, SP - Brazil

**Keywords:** Vascular Resistance, Exercise, Patient Selection, Hypertension, Heredity

## Abstract

**Background:**

A family history of hypertension is associated with vascular and autonomic abnormalities, as well as an impaired neurohemodynamic response to exercise.

**Objective:**

To test the hypothesis that normotensive individuals with a family history of hypertension present an impaired peripheral vascular resistance response to exercise.

**Methods:**

The study included 37 normotensive volunteers of both sexes who were sedentary, eutrophic, and nonsmokers, comprising 23 with (FH+; 24 ± 3 years) and 14 without (FH-; 27 ± 5 years) a family history of hypertension. Blood pressure, heart rate (DIXTAL®), forearm blood flow (Hokanson®), and peripheral vascular resistance were simultaneously measured for 3 minutes during rest and, subsequently, for 3 minutes during an isometric exercise at 30% of maximal voluntary contraction (Jamar^®^).

**Results:**

At rest, the FH+ and FH- groups present similar mean blood pressure (83 ± 7 versus 83 ± 5 mmHg, p = 0.96), heart rate (69 ± 8 bpm versus 66 ± 7 bpm, p = 0.18), forearm blood flow (3 ± 1 mL/min/100 mL versus 2.7 ± 1 mL/min/100 mL, p = 0.16), and peripheral vascular resistance (30 ± 9 units versus 34±9 units, p = 0.21), respectively. Both groups showed a significant and similar increase in mean blood pressure (∆ = 15 ± 7 mmHg versus 14 ± 7 mmHg, p = 0.86), heart rate (∆ = 12 ± 8 bpm versus 13 ± 7 bpm, p = 0.86), and forearm blood flow (∆ = 0.8 ± 1.2 mL/min/100 mL versus 1.4 ± 1.1 mL/min/100 mL, p = 0.25), respectively, during exercise. However, individuals in the FH+ group showed no reduction in peripheral vascular resistance during exercise, which was observed in the FH- group (∆ = -0.4 ± 8.6 units versus -7.2 ± 6.3 units, p = 0.03).

**Conclusion:**

Normotensive individuals with a family history of hypertension present an impaired peripheral vascular resistance response to exercise.

## Introduction

Hypertension is an independent risk factor for cardiovascular morbidity and mortality, affecting approximately 32.5% of the Brazilian population.^1,2^ Therefore, primary prevention has been recommended for individuals at increased risk for the development of hypertension, notably those with a positive family history of the disease.^1^

Studies have shown that normotensive individuals with a hypertensive father and/or mother have an increased risk of development of hypertension.^3-5^ Wang et al.^5^ investigated the impact of parental hypertension on the risk of development of hypertension among 1160 normotensive men during a follow-up of 54 years. In the study, the relative risks of development of hypertension were 1.5, 1.8, and 2.4 among individuals with only the mother, only the father, and both parents with hypertension, respectively, compared with individuals whose parents were normotensive.^5^

The reason for the increased susceptibility to the development of hypertension among offspring of hypertensive parents has not been fully elucidated. However, vascular^6,7^ and autonomic abnormalities,^8-10^ present in this population even before changes in blood pressure level, have been considered relevant in the emergence of this pathology.

In fact, studies have demonstrated an increased sympathetic nervous activity both at rest and during physical exercise in offspring of hypertensive parents when compared with those of normotensive parents.^9,10^ Similarly, it has been observed that individuals with a family history of hypertension have reduced nitric oxide bioavailability^11,12^ and increased endothelin levels^10,12,13^ (endothelial-derived vasodilatory and vasoconstrictor substances, respectively).

During exercise, increased muscle blood flow, which occurs in response to increased metabolic needs, is dependent on vasodilatory mechanisms, especially endothelial and metabolic factors produced in the exercised muscle, which overcome the vasoconstrictor mechanisms.^14^ However, exacerbated vasoconstrictor mechanisms, such as sympathetic hyperactivity, may impair the vasodilatory mechanisms during exercise.^14^ Thus, due to changes in endothelial cells and exacerbated muscular sympathetic nervous activity response present in normotensive individuals with a family history of hypertension, it is possible that the vasodilatory response in this population may be impaired during physical exercise. In this regard, the objective of this study was to test the hypothesis that normotensive individuals with a family history of hypertension have an impaired peripheral vascular resistance response during physical exercise.

## Methods

### Cohort

Based on a sample size calculation using a difference of 2.2 units in peripheral vascular resistance between the means of both groups with and without a family history of hypertension,^15^ standard deviations of 2 units, 5% alpha and 20% beta errors, 14 subjects would be required in each group. Thus, the cohort comprised 37 volunteers, subdivided according to their family history of hypertension among parents in a group with a positive family history (FH+, n = 23) and another with a negative family history (FH-, n = 14).

A positive family history of hypertension was defined as a diagnosis of hypertension in the father, mother or both, evaluated with a questionnaire. A negative family history was defined as the absence of hypertension (blood pressure lower than 140 X 90 mmHg) or a diagnosis of cardiovascular disease in both parents, also evaluated with a questionnaire.

We adopted as the inclusion criteria age between 18 and 40 years, systolic blood pressure below 140 mmHg, diastolic blood pressure below 90 mmHg, and lack of involvement in regular physical exercise for at least 6 months before the study. We did not include individuals with obesity, cardiometabolic diseases, smokers, or receiving treatment with drugs that could interfere with the cardiovascular system, as well as individuals with any bone, muscle or articular impairment that could interfere with the execution of the exercise protocol. We also did not include individuals whose parents had a diagnosis of any other disease besides hypertension.

After prior clarification and agreement, all volunteers signed a free and informed consent form. This study was approved by the Research Ethics Committee in Human Research at HU/UFJF under the number 0119/2010.

### Measures and procedures

#### Anthropometry

To measure the participants’ body mass and height, we used, respectively, a scale with a precision of 0.1 kg and a stadiometer with a 0.5 cm accuracy coupled to the scale (Leader^®^, Brazil). At the time of the evaluation, the volunteers wore light clothes and were barefoot. Body mass index (BMI) was calculated by dividing the participants’ body weight by their squared height (kg/m^2^). Their waist circumference was measured using an inextensible measuring tape (Cescorf^®^) with a 0.1 cm accuracy. All variables above were assessed according to the criteria established by the American College of Sports Medicine.^16^

#### Blood pressure and heart rate

Blood pressure was measured in the right lower limb by the automatic oscillometric method, using a multiparametric monitor (DIXTAL^®^, model 2023).^17^ The heart rate was recorded continuously by five skin electrodes positioned according to the standard lead supplied with the five-way cable of the multiparametric monitor.^17^

#### Forearm muscle blood flow and local peripheral vascular resistance

Muscle blood flow in the forearm was evaluated with the venous occlusion plethysmography technique using the plethysmograph Hokanson^®^ (Bellevue, WA, USA). The volunteer was positioned in the supine position with the nondominant forearm elevated above the heart level to ensure adequate venous drainage.

A silastic tube filled with mercury, connected to a low-pressure transducer and to the plethysmograph, was placed around the volunteers’ forearms, 5 cm away from the humeroradial joint. A cuff was placed around the volunteers’ wrists and another cuff was placed around their upper arms. The wrist cuff was inflated to a suprasystolic pressure level (200 mmHg) 1 minute prior to the measurements and was maintained inflated during the entire procedure. At 15-second intervals, the arm cuff was inflated to a supravenous pressure level (60 mmHg) for 7 to 8 seconds, and then quickly deflated and maintained for the same period of time. This procedure totaled 4 cycles per minute.

The increased tension in the silastic tube reflected the increased forearm volume, indirectly reflecting the increased muscle blood flow in the forearm, and was reported as mL/min/100 mL. The forearm muscle blood flow wave sign was acquired in real time by a computer using the program Non Invasive Vascular Program 3.

The local peripheral vascular resistance was calculated by dividing the mean blood pressure by the muscle blood flow in the forearm, and reported as units.

#### Protocol of isometric physical exercise

To evaluate the responses in blood pressure, heart rate, and forearm muscle blood flow, we used a handgrip isometric exercise protocol using a dynamometer (Jamar^®^, São Paulo, Brazil). Initially, with the volunteer in the supine position, the maximal handgrip isometric strength was calculated as the mean of three attempts of maximal voluntary contraction (MVC) performed on the dominant limb. Hemodynamic measurements were subsequently performed during 3 minutes at rest and, subsequently, during 3 minutes of isometric exercise at 30% of the MVC. 

#### Experimental protocol

The evaluations were performed in the afternoon at the *Hospital Universitário da Universidade Federal de Juiz de Fora* (HU-CAS). The volunteers were instructed not to consume alcohol and/or caffeine or perform vigorous physical activity within 24 hours prior to the evaluations, as well as to not ingest fatty foods on the day of the data collection.

During history taking, the volunteers answered questions related to clinical information about themselves and their parents and underwent anthropometric assessment. After the MVC evaluation, the volunteers rested for 10 minutes in the supine position. After that, we simultaneously recorded their heart rate, blood pressure, and forearm blood flow for 3 minutes during rest and, subsequently, for 3 minutes during the handgrip isometric exercise.

### Statistical analysis

The data are presented as mean ± standard deviation of the mean or as median and interquartile range. To verify the normal distribution of the data, we used the Shapiro-Wilk test. We also verified the homogeneity of variance assumption by the Levene test. Possible differences related to the characteristics of the groups were verified using unpaired Student's *t* test for data with normal distribution, and homogeneity of variance and Mann-Whitney U test for variables violating these assumptions. Sex distribution between the groups is presented in absolute values and percentages. The chi-square test was used to verify a possible difference in sex distribution between the groups.

To test for possible differences between the groups in regards to hemodynamic responses (deltas) during the protocol, we used two-factor analysis of variance for repeated measures (2 X 4 factorial ANOVA, intra- and intersubject; group X exercise time). The Mauchly test was performed and the Greenhouse-Geisser correction was applied in cases in which the sphericity was violated. The main effects and the interaction (group X time) were analyzed with adjustment of the confidence interval by Bonferroni correction. To measure the "effect size," we adopted eta-squared statistics (ɲ^2^), with subsequent classification of its strength according to the values of 0.01, 0.06, and greater than 0.15, as small, medium, and large, respectively.^18^

All statistical analyses were performed using the software IBM SPSS^®^ 20 for Windows (Chicago, IL, USA). The statistical significance was set at p < 0.05.

## Results

The demographic and anthropometric characteristics of the FH+ and FH- groups are described in Table 1. No differences were observed in terms of age, sex, weight, height, BMI, waist circumference, and MVC between both groups. In addition, the groups were similar in regards to systolic blood pressure, diastolic blood pressure, mean blood pressure, heart rate, percentage change in muscle blood flow, and forearm vascular resistance (Table 2).

**Table 1:** Demographic and anthropometric characteristics of the FH+ and FH- groups

Variables*	FH+ (n = 23)	FH- (n = 14)	p value
Age (years)	24 ± 3	27 ± 5	0.09
Male sex (%)	5 (21.7%)	7 (50.0%)	0.07
Weight (kg)	64 ± 11	69 ± 13	0.17
Height (m)	1.67 (1.57 – 1.77)	1.64 (1.47 – 1.81)	0.68
BMI (kg/m^2^)	23 ± 3	24 ± 3	0.24
Waist circumference (cm)	74 ± 9	79 ± 11	0.13
MVC (kgf)	35.4 ± 9.5	41.3 ± 11.4	0.10

Values: mean ± standard deviation of the mean for age, weight, BMI, waist circumference, and MVC; median and interquartile range for height; absolute value and percentage for the male sex; BMI: body mass index; MVC: maximum voluntary contraction.

**Table 2:** Comparisons of hemodynamic variables at rest between the groups FH+ and FH-

Variables	FH+ (n = 23)	FH- (n = 14)	p value
SBP (mmHg)	122 ± 11	121 ± 8	0.69
DBP (mmHg)	64 ± 5	65 ± 5	0.72
MBP (mmHg)	83 ± 7	83 ± 5	0.96
HR (bpm)	69 ± 8	66 ± 7	0.18
MBF (mL/min/100 mL)	3.0 ± 0.9	2.7 ± 0.9	0.16
FVR (units)	30 ± 9	34 ± 9	0.21

Values: mean ± standard deviation of the mean; SBP: systolic blood pressure; DBP: diastolic blood pressure; MBP: mean blood pressure; HR: heart rate; MBF: variation in forearm muscle blood flow; FVR: forearm vascular resistance.

During exercise, the responses in systolic, diastolic, and mean blood pressure, as well as the heart rate and forearm muscle blood flow were similar between the groups. In contrast, during the 3 minutes of the exercise, the forearm vascular resistance decreased significantly only in the FH-group (Table 3). The strength of the effect of the interaction between the factors group and time for this variable was average (η^2^ = 0.10).

**Table 3:** Hemodynamic responses (absolute deltas) during isometric exercise

Variable	Isometric Exercise			F	Interaction effect	η^2^
	1st min	2nd min	3rd min			
**SBP (mmHg)**						
Hypertension+	1 ± 4	16 ± 8*	16 ± 10*	0.201	0.703	0.006
Hypertension -	0 ± 4	7 ± 7*	15 ± 10*			
**DBP (mmHg)**						
Hypertension +	3 ± 3*	9 ± 6*	15 ± 8*	0.234	0.753	0.007
Hypertension -	3 ± 4*	8 ± 6*	14 ± 7*			
**MBP (mmHg)**						
Hypertension +	3 ± 3*	9 ± 5*	15 ± 7*	0.098	0.863	0.003
Hypertension -	2 ± 3*	8 ± 6*	14 ± 7*			
**HR (bpm)**						
Hypertension +	4 ± 5*	9 ± 6*	12 ± 8*	0.169	0.858	0.005
Hypertension -	5 ± 6*	10 ± 7*	13 ± 7*			
**MBF (mL/min/100 mL)**						
Hypertension +	0.5 ± 0.8	0.6 ± 1.0*	0.8 ± 1.2*	1.409	0.251	0.039
Hypertension -	0.8 ± 0.9*	1.2 ± 1.0*	1.4 ± 1.1*			
**FVR (units)**						
Hypertension +	-2.1 ± 4.6	-2.1 ± 5.0	-0.4 ± 8.6	3.777	0.030	0.97
Hypertension -	-7.2 ± 6.4*	-7.9 ± 5.0*	-7.2 ± 6.3*			

Values: mean ± standard deviation of the mean; SBP: systolic blood pressure; DBP: diastolic blood pressure; MBP: mean blood pressure; HR: heart rate; MBF: variation in forearm muscle blood flow; FVR: forearm vascular resistance;

* Significant difference relative to resting (p < 0.05; ANOVA).

## Discussion

The finding of this study indicate that normotensive individuals with hypertensive parents, when compared with their peers with normotensive parents, have a vascular dysfunction characterized by the absence of a decrease in peripheral vascular resistance during physical exercise. It is worth noting that the groups comprised individuals who were sedentary, nonsmokers, and with similar demographic, anthropometric, and hemodynamic characteristics.

Although there are a large number of studies on cardiovascular changes in individuals with a family history of hypertension, we found only one study whose objective was to assess the vasodilatory capacity of this population during physical exercise. This study, conducted by Borghi et al.,^19^ also demonstrated impaired vasodilatory capacity during a handgrip isometric physical exercise at moderate intensity in normotensive participants with a positive family history of hypertension. However, the study did not control for variables influencing the vascular behavior during physical exercise, such as smoking, physical activity, BMI, presence of cardiometabolic diseases, and use of vasoactive medications,^20^ which were controlled for in the present study. Therefore, we have expanded with this study the knowledge about the vascular function during physical exercise in normotensive individuals with hypertensive parents.

In addition, the inability to decrease peripheral vascular resistance among individuals with a family history of hypertension has also been demonstrated at reactive hyperemia peak,^6,7^ a maneuver that despite being triggered by a distinctive mechanism, has a vasodilatory response that correlates with the response triggered by exercise.^21,22^

During physical exercise, the muscle blood flow depends on the balance between dilatory and constrictor forces. In this sense, exacerbation of the sympathetic nervous activity and functional changes in endothelial regulation have been identified as important vasoconstrictor mechanisms responsible for most peripheral vascular resistance observed during exercise in subjects with a history of hypertension.^12^ Indeed, greater muscle sympathetic nervous activity has been reported in offspring of hypertensive parents when compared with those of normotensive parents during handgrip isometric exercise, when directly assessed by microneurography.^9,23^ In addition, we observed increased serum norepinephrine levels both at rest and during handgrip exercise in individuals with a positive family history of hypertension in relation to individuals with a negative history of this pathology.^10^ These factors can explain the results of the present study related to peripheral vascular resistance during exercise.

With regard to the endothelial function, McAllister et al.^11^ observed no differences in endothelium-dependent and endothelium-independent vasodilation evaluated with the dose-response curve induced by acetylcholine and sodium nitroprusside, respectively, among healthy young adults with and without a family history of hypertension. However, these authors verified in offspring of hypertensive parents a vasoconstrictor response mitigated by NG-monomethyl-L-arginine (L-NMMA), an endothelial nitric oxide synthase (eNOS) inhibitor, demonstrating impaired baseline release of nitric oxide in this population. Additionally, Ciolac et al.^12^ evaluated women with a family history of hypertension and observed, both at rest and during a maximal incremental treadmill test, reduced levels of nitrite and nitrate, the end products of degradation of nitric oxide, which also suggests a reduction in the production/bioavailability of this important vasodilator.

In addition to reducing the bioavailability of nitric oxide, increased levels of endothelin, an endothelium-derived vasoconstrictor substance, have also been observed in offspring of hypertensive parents when compared with offspring of normotensive parents, both during handgrip exercise,^10,13^ as well as during incremental exercise test on a treadmill.^12^ Therefore, it may be hypothesized that the increased vascular resistance observed in offspring of hypertensive parents during exercise may be related, at least in part, to a reduced endothelial production of vasodilatory substances and increased production of vasoconstrictor substances.

Declines in vascular function are associated with the development of atherosclerosis and future cardiovascular events.^24^ Moreover, with the increased sympathetic tone, vascular dysfunction is involved in the development of hypertension and may be related to the greater predisposition of offspring of hypertensive parents to developing this disease.^12^

In this study, the responses in blood pressure and heart rate during exercise were similar between the groups. In addition, the groups presented a physiological increase in these variables throughout the test. Our results reproduce the findings of other authors,^9,10,25^ who also observed similar responses in blood pressure and heart rate during handgrip physical exercise. On the other hand, the study by Greaney et al.^23^ observed an exacerbated response of the mean blood pressure during exercise in young women with a positive history of hypertension. The different results found may be related to the characteristics of the study population. The sample in the present study comprised sedentary individuals of both sexes, whereas the sample in the study by Greaney et al.^23^ comprised sedentary and active women. It is worth noting that the studies investigating blood pressure levels during physical exercise involving large muscle groups, such as exercise on a cycle ergometer^26,27^ and knee extension isokinetic exercise,^8^ have observed increased blood pressure levels in offspring of hypertensive parents, suggesting that the increased amount of muscle mass involved could be related to the cardiovascular hyperreactive responses observed in this population during these types of exercises.

This study showed that healthy young individuals without cardiovascular risk factors besides a family history of hypertension have impaired vasodilation during exercise. The increased peripheral vascular resistance during physical exercise may explain, at least in part, the blood pressure hyperreactivity in normotensive individuals during physical exercise. It has been documented that the exacerbated blood pressure response during exercise stress testing associated with increased total peripheral vascular resistance^28,29^ is a prognostic factor for cardiovascular events and mortality in middle-aged men^30^ and hypertensive individuals,^29^ in addition to being related to cardiac remodeling in pre-hypertensive individuals.^28^ However, until the present moment, there have been no longitudinal studies designed with the intention of investigating the prognostic application of the vascular behavior in offspring of hypertensive parents during physical exercise and the possible development of hypertensive disease.

Thus, the results of this study emphasize the importance of a preventive intervention with measures aimed at reducing vascular resistance and, consequently, acting in the prevention of hypertension in this population. In this regard, physical exercise has been implicated as an important strategy for prevention of hypertension in offspring of hypertensive parents,^3^ considering the beneficial results of training on pathophysiological factors involved in the emergence of this pathology, such as the sympathetic hyperactivity and vascular dysfunction,^31^ which are often present in susceptible individuals, even before the increase in blood pressure levels. 

### Limitations

This study has some limitations that should be mentioned. The diagnosis of hypertension in the volunteers’ parents was reported by the volunteers themselves (self-report). Although this information has been self-reported in several studies,^6,23,32^ future research should include a detailed medical assessment of the parents. In addition, the women in this study were not evaluated during the same period of the menstrual cycle, a fact that could also configure a limitation of this study. However, Jarvis et al.^33^ and Carter et al.^34^ found no influence in young women of the ovarian cycle phase on sympathetic nervous activity, heart rate, and blood pressure during handgrip exercise and mental stress, respectively.

## Conclusion

We conclude that young normotensive individuals with hypertensive parents have impaired vasodilation during isometric physical exercise.
